# AGE, FUTURE TIME PERSPECTIVE, AND EVERYDAY PHYSICAL ACTIVITY AND NUTRITION IN COUPLES POST STROKE

**DOI:** 10.1093/geroni/igz038.3492

**Published:** 2019-11-08

**Authors:** Elizabeth Zambrano Garza, Theresa Pauly, Wolfgang Linden, Maureen C Ashe, Rachel Murphy, Kenneth Madden, Christiane A Hoppmann

**Affiliations:** 1 University of British Columbia, Vancouver, British Columbia, Canada; 2 University of British Columbia, Vancouver, Canada

## Abstract

Physical activity and fruit/vegetable consumption are recommended to help prevent and manage cardiovascular disease. Yet, most people struggle to meet physical activity and nutrition guidelines. This study examined the role of age and future time perspective for these two health behaviors using repeated daily life assessments as well as accelerometry-based step counts from 70 persons living with the effects of stroke and their partners (50% female, M age=69 years). Consistent with previous research, older age and living with stroke were associated with taking fewer steps in everyday life but also with consuming more fruit and vegetables. Furthermore, participants who viewed their future as being filled with many opportunities took more daily steps and ate more fruit and vegetables than participants low in future opportunities. Further analyses will examine dyadic associations in these two health behaviors as well as partner factors that may facilitate or hamper the engagement of the behaviors. It is important to analyze these relationships to gain further insight into the effects partners have on each other.

